# Clear skies ahead: optimizing the learning environment for critical thinking from a qualitative analysis of interviews with expert teachers

**DOI:** 10.1007/s40037-019-00536-5

**Published:** 2019-09-27

**Authors:** Lynn E. Jaffe, Deborah Lindell, Amy M. Sullivan, Grace C. Huang

**Affiliations:** 1 Department of Rehabilitation Sciences, Florida Gulf Coast University, Fort Myers, FL USA; 2 Frances Payne Bolton School of Nursing, Case Western Reserve University, Cleveland, OH USA; 3 Carl J. Shapiro Institute for Education and Research, Harvard Medical School, Beth Israel Deaconess Medical Center, Boston, MA USA; 4 Department of Medicine, Beth Israel Deaconess Medical Center, Boston, MA USA; 5 Program in Medical Education, Harvard Medical School, Boston, MA USA

**Keywords:** Learning environment, Critical thinking, Qualitative research

## Abstract

**Introduction:**

The learning environment refers to the physical, pedagogical, and psychosocial contexts in which learning occurs and critically influences the educational experience of trainees in the health professions. However, the manner in which individual faculty explicitly organize the educational setting to facilitate learning of essential competencies such as critical thinking deserves more examination; lack of attention to this component can undermine the formal curriculum. The purpose of our study was to examine how faculty shape the learning environment to advance their learners’ development of critical thinking.

**Methods:**

We took a constructivist grounded theory approach using the framework method for qualitative content analysis. Data were derived from interviews conducted with 44 faculty identified as skilled teachers of critical thinking at eight academic health professions institutions.

**Results:**

Three major themes emerged regarding participants’ descriptions of their experiences of how they optimized the learning environment to support critical thinking: 1) *Setting the atmosphere* (establishing ground rules, focusing on process rather than answers, and building trust), 2) *Maintaining the climate* (gently pushing learners, tolerating discomfort, and adjusting to learner level), and 3) *Weathering the storm* (responses to challenges to learning critical thinking, including time and effort, negative evaluations, and resistance to effortful learning).

**Discussion:**

An optimal learning environment for critical thinking was actively created by faculty to establish a safe environment and shared understanding of expectations. Understanding how to produce a conducive learning climate is paramount in teaching essential topics such as critical thinking. These findings have potential utility for faculty development initiatives to optimize the learning environment.

**Electronic supplementary material:**

The online version of this article (10.1007/s40037-019-00536-5) contains supplementary material, which is available to authorized users.

## What this paper adds

While consideration of the learning environment is a vital ingredient to how students learn skills, the literature has not specified how expert faculty actively shape the learning environment in daily teaching interactions to promote critical thinking. Results of our multi-centred qualitative analysis of interviews with faculty across the health professions revealed concrete strategies to create a learning environment necessary to support higher thinking skills. These practices include setting ground rules to establish safety and respect, the ability to push learners for deeper understanding, tolerance of disagreement, and the willingness to weather challenges such as learner resistance.

## Introduction

The learning environment is defined as ‘the physical, social, and psychological contexts in which [learners] learn and grow professionally’ [1]. It is a major contributor to the educational experience of learners within the health professions. What occurs in the formal curriculum (e.g., didactic materials, classroom instruction aligned with learning objectives) can be easily weakened if it lacks a conducive milieu for new knowledge, skills, and competencies to take root.

However, the focus on the learning environment as a critical part of the hidden curriculum [2–5] has generally emphasized its negative, largely unintentional aspects such as poor role modelling and insufficient curricular focus on particular topics. How individual faculty engineer the learning environment in a positive way to enhance the formal curriculum has received less attention.

One fundamental learning objective of the formal curriculum is that learners develop a capacity for critical thinking in a clinical context [6–8]. Critical thinking has been defined as ‘the ability to apply higher cognitive skills (e.g., analysis, synthesis, self-reflection, perspective-taking) and/or the disposition to be deliberate about thinking (being open-minded or intellectually honest) that leads to action that is logical and appropriate’ [9]. Critical thinking is arguably a cornerstone for health professionals in delivering patient care and is relevant for faculty-learner interactions during decision-making. Thus, it offers an entryway into examining how faculty shape the learning environment when teaching this important competency.

We conducted a multi-institutional qualitative study of faculty’s perspectives of teaching critical thinking [10]. Our overarching research question was: How do faculty identified as skilled teachers of critical thinking characterize their teaching? Here we describe their views on how they characterized the hallmarks of the learning environment necessary to support critical thinking and the required skills to create this climate, an investigation that has not been previously pursued. These findings have direct relevance for efforts addressing how the learning environment can be enhanced by faculty to foster the teaching of core clinical skills and competencies.

## Methods

This multi-site, qualitative study was part of a larger effort to characterize strategies for teaching critical thinking in health professions education in the US, for which we conducted semi-structured interviews of 44 faculty at eight schools of medicine and nursing, as introduced in a prior publication [10]. We used a constructivist grounded theory approach, in which researchers attend to the active co-construction of knowledge and meaning in both the collection and analysis of data [11, 12]. We chose a grounded theory approach as it is appropriate for developing detailed, nuanced characterizations of a particular social/educational process (here, creating learning environments to foster critical thinking) based on the interpretations of those directly involved in it [13]. Thus our in-depth, semi-structured interviews allowed faculty to elaborate on the context, process, meaning, and challenges of their experiences in structuring environments to promote critical thinking among learners.

At the time of the interviews and to date, our research team (LJ, DL, AS, and GH), respectively, were faculty in occupational therapy, nursing, and medicine, and held the following credentials, ScD, DNP, EdD, and MD. All authors were leaders in health professions education at three of the study institutions for more than 20 years, with at least 10 years of experience with qualitative research and investigators of critical thinking for at least 8 years. We were equally involved in all aspects of the project, including study design, interviewing, and analysis.

### Study site and participant recruitment

We used a purposive-snowball approach to recruit participants. Eight medical schools (Case Western Reserve University, Dalhousie University, Medical College of Georgia at Georgia Regents University, Harvard Medical School, Indiana University, Pennsylvania State University, the University of Massachusetts, and the Weill Cornell University) had been selected to participate in the Millennium Conference on Critical Thinking [9] and agreed to take part in multi-institutional initiatives after the conference. Beth Israel Deaconess Medical Center was the lead site, and members of a post-conference task force on teaching critical thinking agreed to be site coordinators at each of the eight institutions. The site coordinator obtained exemption or approval, as indicated, for research activities through the Institutional Review Boards (US) or Research Ethics Board (Canada) of their institutions.

The site coordinators sent a standardized email to clerkship, course, program, and residency leaders seeking nominations of faculty at their institutions considered exemplary teachers of critical thinking, i.e., teachers with reputations for teaching critical thinking who had won teaching awards or who directed clinical reasoning and other related courses. The email contained a description of the study purpose and consent form. Those who agreed to take part were directed to an online pre-screening survey regarding critical thinking. To identify interviewees, responses to the survey were systematically reviewed by two task force members using a rubric focused on three domains—alignment of critical thinking definition with the study definition, substantive content, and quality of response. In all, respondents nominated by peers and meeting all three inclusion criteria via the screening survey were invited to participate in a single interview.

### Data collection

We used a semi-structured interview guide developed and iteratively revised by members of the critical thinking task force and piloted with volunteers not involved in the study. The interview script can be found in the online Electronic Supplementary Material. One author (GH) coordinated interview assignments to ensure that authors did not interview faculty from their own institutions. Prior to the interview, each participant received the consent and our study definition of critical thinking. The interviews were conducted by phone, at a date and time convenient for the participant, with only the interviewer and participant on the call, and began with introductions of the interviewer and a restatement of the consent. The participant was invited to ask further questions about the study and the role of the interviewer. The calls lasted up to 30 min.

### Data analysis

Data management and content analyses were concurrent with data collection and conducted according to the principles of the framework method. The framework method is part of the ‘broad family of analysis methods often termed thematic analysis or qualitative content analysis … Its defining feature is the matrix output: rows (cases), columns (codes) and “cells” of summarized data, providing a structure into which the researcher can systematically reduce the data, in order to analyse it by case and by code’ [14]. The framework method accommodated our study purpose and methods as it was developed for use with interprofessional teams and designed to include analytic approaches that are both deductive (exploring data using predetermined categories from literature, prior research, or the participants—here, strategies to teach critical thinking [10]) and inductive (identifying emergent themes from the data—here, how the learning environment can support teaching of critical thinking). The framework method includes seven stages: 1) transcription, 2) familiarization with the interview, 3) coding, 4) developing a working analytical framework, 5) applying the analytical framework, 6) charting data into the framework matrix, and 7) interpreting the data [14, 15].

Implementation of Stage 1 included two parts: A) the interviewers typed notes during the interview into an online form, and B) the interactions were audio-recorded and transcribed by a third party verbatim such that participants could not be identified. In Stages 2–7 of the framework method, all four authors analyzed the dataset using a systematic, step-wise process that led to an analytical framework composed of codes derived from iterative observations from the data until thematic saturation was reached. The codes were associated with definitions and grouped into categories. Aided by Dedoose™ qualitative software, the analytic framework was then applied to the entire dataset. We then organized the categories into themes as depicted in the model described below. During each phase of analysis, transcripts were reviewed individually by pairs of authors who then discussed and reached consensus regarding their results. Similarly, all four authors discussed and reached consensus on their results for each stage of analysis. Analytic memos were used by team members to document and communicate their thinking as they studied the transcripts. During phase one, a major set of themes related to the learning environment arose at a frequency and depth that compelled a second phase of the study with separate analysis and report centred on the learning environment. For this second phase of the study, we report on the identified codes pertaining to the teaching/learning environment and used the framework method as described above.

### Trustworthiness

The following strategies were implemented to ensure the results of the study demonstrated characteristics of trustworthiness, namely, credibility (truth value), transferability, dependability, and confirmability: 1) sufficient number and breadth of interviews (phase one) and dataset (phase two) of sufficient number and breadth of teaching/learning settings to allow a deep understanding of how the participants teach critical thinking and full implementation of the framework method, 2) peer debriefing through bi-weekly phone conferences and electronic sharing of analytic memos, 3) engagement by each member of the four-person research team in the implementation of all stages of the framework method, review of 25 transcripts by all four raters comparing the coding and defining any disputed codes more clearly, with inter-rater reliability testing with paired coding teams on 53 excerpts, 4) detailed description of the participants and extensive use of direct quotes to illustrate codes, categories, and themes, 5) a ‘paper trail’ using thorough, de-identified records of all aspects of the study easily accessed by team members on a secure, de-identified, document-sharing site, and 6) member checks during phase one, by asking each participant to review and comment on a summary of the results [16]. No revisions were suggested in response to the member checks.

## Results

The online screening survey was begun by 291 medical and nursing faculty. Sixty met the inclusion criteria and were invited to participate in the study. Excluding non-respondents, 44 faculty members underwent interviews, with an overall response rate of 73% of eligible faculty members (ranging 29–100% across eight institutions).

Forty participants were MDs, one was a nurse, and three were PhDs. These teaching faculty held additional leadership roles, such as serving as course, program, or clerkship directors. The teaching settings ranged from small group to large group classrooms and to a range of clinical locations. The learners taught by these faculty spanned the spectrum of healthcare professional training from pre-licensure nursing to clinical fellows in medicine.

**Fig. 1:**
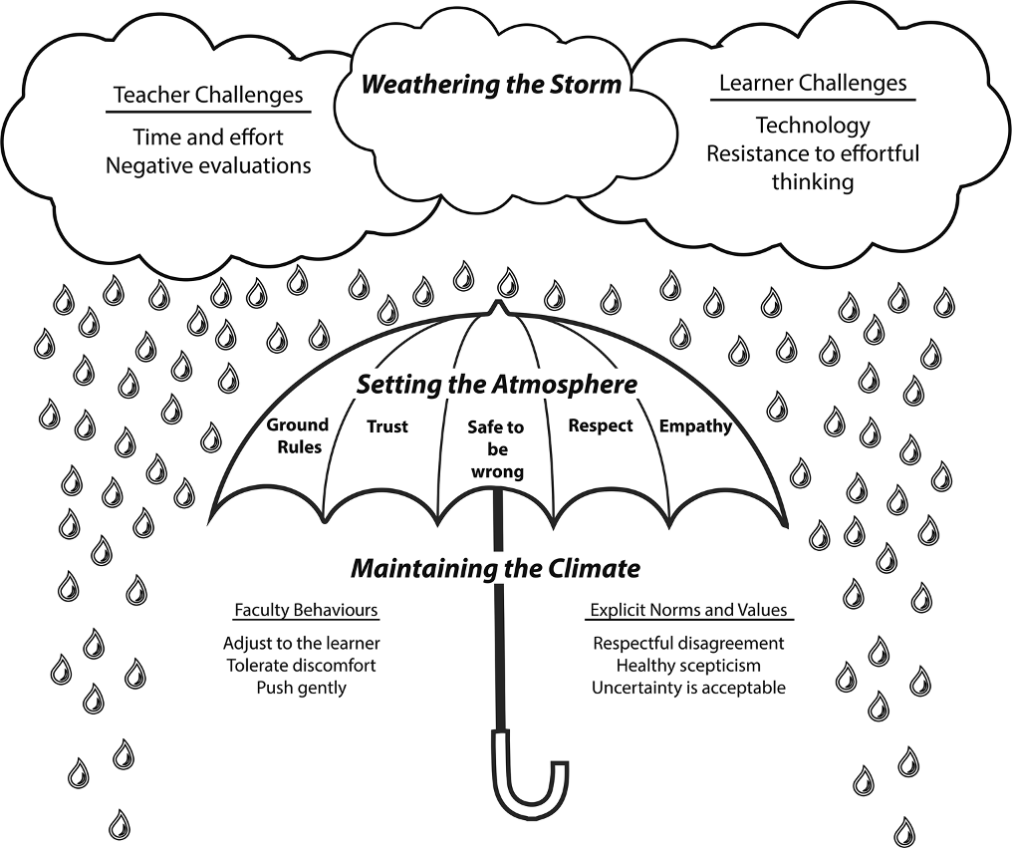
Sheltering against the teacher-learner challenges that threaten the learning environment

Faculty descriptions of the learning environment revolved around three major themes: 1) Setting the atmosphere, 2) Maintaining the climate, and 3) Weathering the storm. Instead of in-line text, we present all illustrative quotes for our findings, categorized by theme in Tab. 1, which can be found in the online Electronic Supplementary Material. We also synthesized all the themes into a pictorial representation of the learning environment, inspired by some of the language used by interviewees and applying a metaphor befitting the topic (Fig. 1).

**Table 1:** Faculty descriptions of essential elements of learning environments to foster critical thinking. Results of qualitative analysis, organized by theme, with subtheme definitions and supporting quotes

Theme	Description	Example quotes
*“Setting the atmosphere”: Creating a safe environment to facilitate CT* This theme relates to faculty descriptions of establishing a safe environment		
Ground rules	Discussing expectations, values, and norms with learners at the start of a classroom or clinical experience	I think it starts off initially with some groundwork setting the atmosphere. Whether that’s a classroom setting, or lab, or clinical, students and faculty … everyone … needs to feel … respected. Their opinions are important. The classroom … needs to be a setting where people feel relaxed and safe … students get a lot of positive feedback and … see that it’s okay to make mistakes or think out of the box, to be creative. S2F16
Building trust	Statements indicating that feedback is intended to support learning and not be shaming	It’s a supportive learning climate with positive role modeling rather than shame-based or criticism-based, overly critical. It’s critical in a healthy sense … critical without being overly critical; thinking without overthinking; feeling without overreacting. Those kinds of things are really important. S3F40
Safe to be wrong	Explicit statements about importance of learning and thought process rather than having the “right answer”	So you remove the intimidation factor. In other words, you can make all the mistakes you want. No one is going to be hearing it. I tell them ahead of time, “I’m not looking at your answers. I’m never going to see them. That’s not what the discussion is about. It’s about what you learn.” S4F10
Respect	Modeling and/or explicitly stating the value of respect	I’m really explicit that I really could care less about what their conclusion is. I care about the argument that they make to support their conclusion. That’s important … for establishing the safety of the environment. S7F10
Empathy	References to empathy in the teacher-learner or learner-learner relationships	I think empathy is [important]—and just as it is another reason why the doctor-patient interaction is very similar to the attending-learner interaction. S8F39
*Maintaining the climate: Behaviors and explicit norms and values that sustain the environment for CT* Specific faculty behaviors, norms, values, and expectations of learners for maintaining a supportive environment		
Adjust to learner level	Faculty behaviors related to adjusting their teaching to the learner’s level of training and receptivity	If they’re so early in their knowledge base that the challenge for them is just simply to put the knowledge base in … then you can’t challenge that knowledge base; they’re busy still acquiring it. S6F9
		If I have different levels of learners, we’ll move upstream starting with the most junior learner, asking some questions and then move on to somebody who’s a little more senior … trying to keep everybody engaged. If you get the sense that someone is intimidated, then you obviously try to make it easy for them, so again, the engagement is not one that’s intimidating or something that’s going to be a fearful experience for them. S8F44
Push	Faculty behaviors of gently pushing, inviting, encouraging, or nudging learners for ideas (includes descriptions of explaining this explicitly to learners)	I have the pictures of the students in front of me. I think it helps to … use their names and to show investment that, “Okay, I heard you. This is what you said, thank you Carol. Josh, can you take Carol’s point one step further?” Sometimes they need that, sometimes they don’t … Sometimes it really does take an invitation to the conversation. S8F15
		Then the other thing that I do, and I haven’t seen anything about this in the literature, but I think it helps, is, and I introduce it to my students actually, I warn them, I say, “I’m gonna ask you ‘why’ a lot, and it’s gonna irritate you.” S7F10
		What I feel like I’m trying to do in teaching is do that sort of gentle nudging and highlighting of other possible perspectives and encouraging folks, even if they don’t necessarily agree with what’s being presented, to interrogate and think about that disagreement and to be more thoughtful or specific about what it is that they find challenging or wrong or problematic about whatever position it is that I’m presenting. S3F5
Respectful disagreement	Establishing norms of disagreeing with respect and engaging honestly about differences	My experience has been that … you start slowly and build up the trust, the relationship, the safe space that you mentioned earlier for having these sorts of discussions and sort of getting the group to a point where they can have really fruitful disagreements with each other, but also with me as well, and for there to be that … respectful back and forth. S3F7
		Some of it is at the outset of the tutorial for me being explicit about the … training that I come from … that expressing disagreement and challenging people is how you express respect, and the worst thing you could do to someone is just sort of say “Oh, that’s nice” to their paper and move on. It’s really that engagement that shows that you think that someone and their thinking is worthwhile. S3F5
Healthy skepticism	Faculty promoting learner stance of skepticism and not accepting information at face value	I’d just like to reinforce that I think critical thinking embodies skepticism; it embodies judgment about reliability of data and incorporates that into final judgment-making; that those are the sorts of things that a competent health professional should, I believe, bring to their approach to their profession. That’s personal, but I think a healthy dose of skepticism is a good way to encourage people to think closely and carefully about how they approach the information they have at their disposal. S3F14
Uncertainty is acceptable	Faculty advocating for learner cognitive recognition and acceptance of uncertainty	The work that we do in ethics emphasizes that there is always uncertainty and that context matters and things are gray as opposed to black and white. I think it’s something that I try and tie into other uncertainties that they might experience and sort of say that it’s not just about the ethics, but it’s about medicine and human bodies and complexity and things like that. S3F5
		You can’t put it all together and yet they need to have the confidence and the ability to … make best guesses or to deal with unknowns … I think that’s also really a part of critical thinking that’s important and difficult for students sometimes. S8F5
Tolerance for discomfort	Faculty supporting student (and faculty) affective aspect of tolerating the discomfort of not knowing or struggling with new ideas	One of my theories about critical thinking is that it requires a certain amount of acceptance and tolerance of emotional discomfort, on the part of the student, as well as on the part of the teacher. I think the teacher has to be willing to tolerate the discomfort of asking questions that will challenge a student and allow them to kind of be challenged … Then the student has to have the capacity to … tolerate the not-knowing or the not-being-sure and kind of having to think out loud or to risk being wrong. S4F27
		I think if you put someone in a situation that maybe isn’t completely comfortable, because … maybe they’re not as prepared for that particular question, but they tend to learn from that, and really retain that information. S6F38
		Part of what I do is encourage and promote that kind of emotional willingness … and acceptance of that sort of discomfort as part of the process. I try to give my students permission to be wrong—to try and fail—to take small steps in the direction that they sort of want to go in terms of their learning, even if it’s not perfect. S4F27
*Weathering the storm: Challenges that arise for faculty and learners*		
Time & effort	Faculty and learner time and effort required	I have noticed that as time becomes more a factor … toward the end of the course one is more inclined to just give a straight answer; but that reinforces the student’s wish, whatever the background of students, simply to memorize … Now it’s very time-consuming to do that … but I’d rather have them work through it than to have them simply told that that is what it is and go away and look it up. S3F14
		I see that more and more … “Well, I can just find [it] on the web” … They get frustrated. … I think it causes their brains to hurt a little bit because a lot of them are like, “Where’s the easy answer? Well why won’t you just tell me?” S2F7
		Now it’s very time-consuming to do that …, and students … sometimes are not tolerant of being treated in that fashion, but I’d rather have them work through it than to have them simply told that that is what it is and go away and look it up. S3F14
Negative evaluations	Pushing students to think can result in negative evaluations from learners	I’ve mentioned the feedback tends to vary from very positive to occasionally very negative. I think if I was looking for a technique that would win the teacher the popularity stakes, I probably wouldn’t go for critical thinking. S3F37
		I don’t always have great evaluations. Some students don’t—I tend to push this—not this concept, but I push students to think. I want to push students to think. I’m very passionate about it. Some students don’t react well to that. There’s always the few that are—think of it as me being mean or critical, but it’s not. It’s honestly only to help them and help them evolve as a professional nurse. S2F7
Learner challenges: Technology shortcuts	Technology providing superficial information and limiting learners’ thinking	I believe the electronic medical record has not facilitated clear medical communication. We get lots of words but it’s not very information-dense and there’s a tendency to copy and paste, for instance, the assessment … should have evolved and changed by now, but it’s like a fly in amber: it’s frozen; it’s not changing. S7F13
Learner challenges: Resistance to effortful learning	Students not wanting to think through issues	If they give answers such as, “Because that’s the way we always do it”, or it’s very obvious that they’re just trying to regurgitate some information that they know, but they’re not really applying the info, I can usually pick up on it pretty quickly. Usually, by their answers I can tell if they’ve thought through the alternatives, and/or if they just said an answer that they thought that that’s typically how we do it, and that’s why we should do it, in that case. S6F38

Respondent number indicates school number (S1–S8) and individual faculty ID (F1–F44)

### Setting the atmosphere: Creating a safe environment

Establishing a backdrop from which critical thinking could be explicitly developed and demonstrated coalesced on the primary theme of ‘safety’. As one faculty member put it, an essential first step was ‘*starting off initially with some groundwork setting the atmosphere*’ so ‘*people feel relaxed and safe*’ (S2F16). Participants described a safe atmosphere as a supportive learning environment in which students had permission to explore without the fear of being wrong, were encouraged to take care of each other, and felt respected. Safety allowed student groups to have fruitful disagreements and develop skills in argumentation while they wrestled with answering questions, exposing their own gaps, and assessing their own thinking. Strategies that contributed to creating a safe learning environment included building trust by establishing ground rules and structure for learners at the outset, providing clear expectations and encouraging teamwork and empathy during problem-solving. In addition, focusing preferentially on the process of deduction and decision-making, rather than purely on answers, was identified as an attribute of a safe environment. These features were reported as necessary for a student to develop the reasoning and reflection required for critical thinking.

### Maintaining the climate: Behaviours, norms and values that sustain a learning environment for critical thinking

This second overall theme reflected participants’ recognition that the learning environment needed to be sustained and developed by faculty behaviours, explicit norms, and values. To that end, one ability important for teaching critical thinking was being able to scaffold teaching according to where learners were in their critical thinking development. Those more advanced in knowledge, critical thinking skills, or training were felt to be more likely to handle some of the intellectual discomfort that critical thinking can produce. These students could then model critical thinking for the newer learners.

Many respondents also focused on the ability to push students into the range of desirable difficulty to elicit critical thinking without threatening safety. In addition to ‘*push*’, the words used to address this theme included ‘*encouraging*’, ‘*inviting*’, or ‘*nudging*’. They specified two facets of skill on the part of the teacher—an active component, where the faculty member learned to inquire deeply, and a receptive component, where the faculty member tolerated the discomfort of uncertainty and challenge that was a common attribute of the learning environment. The latter required faculty to wait out silences and refrain from spoon-feeding answers. Also, the underlying assumption for the need to nudge or push was that critical thinking teaching required effort, discipline, and some risk and thus might not arise naturally from learners.

Faculty further described the benefits of teaching critical thinking within an environment that liberated ideas and decreased nervousness and fear. This included guiding student groups to have fruitful disagreements and develop skills in argumentation while they wrestled with answering questions, exposing their own gaps, and assessing their own thinking. Maintaining this safe learning environment helped students develop security in expressing themselves, develop a sense of a healthy scepticism, and enhance their comfort with uncertainty.

### Weathering the storm: Challenges that arise for faculty and learners

The third overall theme referred to challenges participants encountered in teaching critical thinking and the potential faculty or learner responses that could impact, or be impacted by, the learning environment. For some participants, time and energy expended in planning and structuring opportunities to elicit critical thinking were extensive and sometimes had to give way to expediency. Faculty acknowledged their approaches in teaching critical thinking did not always lend themselves to externalities such as positive teaching evaluations. However, some indicated that cultivating effective critical thinkers justified the work and occasional frustration, as they described situations when time and effort resulted in epiphanies of knowledge, in a tone that suggested they derived vicarious satisfaction as well.

The faculty also articulated learner-related challenges to teaching critical thinking. The easy availability of information on the Internet, for instance, presented a constant temptation to shortcut critical thinking. Similarly, the cognitive work involved in critical thinking resulted in some learner resistance to effortful thinking. Furthermore, many faculty described differences in student openness toward learning critical thinking; in some cases, distinctions were portrayed as bimodal and easy to identify from the outset. In all, faculty expressed a willingness to confront learner struggles with critical thinking (and the attendant secular influences that may thwart it) at the possible professional cost to themselves.

## Discussion

In our multi-institutional, qualitative interview study, the learning environment emerged as a central element in faculty narratives about how to foster critical thinking among learners. In multiple, nuanced ways, faculty described how they established safe venues in which learners could grapple with uncertainty and risk. Faculty emphasized the importance of establishing a safe environment early in their course or clinical work with learners to establish a sense of trust, respect, and empathy among learners and between learners and faculty. Prominent in many faculty narratives were descriptions of routines of ‘setting the atmosphere’ of a new teaching experience by explicitly talking with learners about the importance of making their thinking visible and verbalizing the rationales for their decisions—rather than striving to provide a ‘right’ or ‘wrong’ answer. In describing how to sustain and develop a conducive learning climate, respondents highlighted important skills such as the ability to probe learners’ thinking, to tolerate some measure of learner (and their own) discomfort, to recognize the level of student knowledge, and to help students move through their resistance to learning independently. Quotes from these individuals selected for their teaching aptitude powerfully gave voice to their commitment and skill in crafting a learning environment that promoted healthy discourse without threatening learners’ self-esteem. Our findings have generalizable value for faculty development efforts to optimize the learning environment.

These faculty narratives demonstrated that cultivation of a favourable learning environment took significant effort, skill, and experience. Our findings suggest that faculty educators can and do recognize the influence of the environment on learning, understand how to perform a needs assessment that transcends simple identification of learners’ knowledge gaps, and know where to place learners on the spectrum of ability in critical thinking as well as foresee their potential growth. Additionally, faculty pointed to the need for facility in pushing learners past ingrained thinking habits, and self-regulation to dynamically assess the learners’ responses, and knowing when to step back. Given the evident skill and effort depicted in these practices, our findings suggest that incorporation of teaching scripts needed to accomplish all these goals would require deliberate skill development, potentially with hands-on methods such as role playing and direct observation.

Our results also called attention to the investment of time that teachers need with learners to establish environments conducive to learning. Critical thinking does not develop instantaneously; it is a cognitive habit that matures over time in learners, especially in those who may be initially resistant. Ideal learning environments are situated within meaningful interpersonal connections, and time allows the essential relational elements of trust, respect, and empathy to deepen. It is difficult to imagine how optimizing learning environments can occur without longitudinal teacher-learner relationships. Therefore, efforts to address learning environment concerns may require restructuring of curricula (e.g., longitudinal integrated clerkships in undergraduate medical education [17]) and/or teaching assignments.

Last, the dedication of these faculty to explicitly tackle the learning environment demonstrated resolve and commitment. These interviews provided an intimate window into the experiences of faculty as they confronted both successes and failures in working with learners to think critically. They reported a willingness to sacrifice popularity and productivity in order to motivate their learners in ways countercultural to an era of instant gratification. This commitment also comes at a time when faculty face overwhelming clinical and academic pressures and may be intensely attuned to institutional concerns about learner mistreatment [18, 19]. Faculty willing to confront these challenges of the learning environment may benefit from direct observation, feedback, and affirmation.

A Macy Foundation conference comprehensively addressed at a systems level the components of optimal learning environments for the health professions [20] and a robust body of medical and nursing literature has illuminated a multitude of perceptions and influences on the learner environment [21, 22]; these works shed light in ways that align with our findings. Several studies have examined aspects of faculty interactions that foster a positive learning environment, including attention to the psychosocial context [23, 24], faculty attitudes [25], and expectation-setting [26]. Safety has been described as a paramount feature of the learning environment [18, 27]. How to navigate the thorny balance between challenging learners in a non-threatening way has been described to a limited extent [28–31]. These studies all derive importantly from the perspective of the trainee or of the institution as a whole. However, to our knowledge, no previous work has captured how individual faculty actively configure the learning environment to support clinical teaching.

A limitation of our work is that our selection was not representative of all faculty and may not apply to those who do not actively teach critical thinking. Non-physician health professionals were underrepresented in our sample. Also, we made no direct measurements of the learning climate [32–34] described in these interviews to verify whether safety and other attributes did indeed promote critical thinking. We did not ask how long faculty had been working with the learners in question nor did we have sufficient details about the teaching setting specific to each situation to draw conclusions about which strategies worked in particular settings. We did not directly assess other vital, faculty-driven aspects of the learning environment that may impact critical thinking, such as communication or professionalism [3]. By nature of our focus on faculty, our contribution to the literature on the learning environment focuses only on the teacher-learner dyad and does not directly address learner-learner issues, clinician-clinician interactions, nor institution-teacher factors such as decision support systems. Though the intensity of our data collection and analysis lends credence to the notion that an optimal learning environment is critical, we recognize through our prior work that efforts to create a conducive environment must be combined with explicit efforts to teach critical thinking. In short, a good learning environment is necessary but not sufficient.

Our study has implications for the continuing professional development necessary to support learning climate reform at health professions institutions. Programs can build on current efforts to address the hidden curriculum [5] and design curricula to maximize meaningful interactions between faculty and learners and each other but can also draw attention to concrete skill building, as mentioned earlier. These efforts may need to vary with the learner’s level of training and, based on our work, novices may require more explicit attention to the learning environment than more advanced beginners. A research agenda should include an examination of critical thinking learning environments specific to medical students, who are in a more formative yet vulnerable time of training and may need especial attention to ‘setting the atmosphere’. Future work should also entail a broader representation of the health professions to increase generalizability. In short, we believe this work to inform broad-based efforts to optimize the clinical learning environment so that health professions trainees thrive while developing mastery in core competencies.

## Caption Electronic Supplementary Material

Interview script
