# Supplementary material for: Clear skies ahead: optimizing the learning environment for critical thinking from a qualitative analysis of interviews with expert teachers
Source: Perspect Med Educ. 2019 Sep 27;8(5):289–97. doi: 10.1007/s40037-019-00536-5 (PMC6820647; doi:10.1007/s40037-019-00536-5)
Supplement: Supplementary file 1 — Interview script [file 40037_2019_536_MOESM1_ESM.doc]

Interviewer name:

Date of interview:

Participant ID number:

**Introduction**

Thank you very much for agreeing to participate in this interview. As you may know, you were nominated by one of your colleagues as someone who is exemplary in teaching critical thinking.

The purpose of this interview is to derive best practices in teaching critical thinking from faculty members involved in the training of physicians, nurses, and other healthcare professionals. This is the work of a national task force that was the outgrowth of the Millennium Conference on critical thinking, which took place in Boston in May 2011. The results will be presented in a manuscript submitted for publication in a journal and may spur further initiatives in critical thinking.

I will be asking some questions about your background and your teaching activities. I will then ask you about teaching strategies, methods, and tools you use to teach and to promote critical thinking. The entire interview should take about 15 minutes.

You have received the written consent form by email, and I will now ask for your verbal consent. Verbal consent will entail that you: 1) have received the written consent form here, 2) agree to participate in this study, 3) acknowledge the right to withdraw at any time, and 4) permit the use of de-identified quotations in the ultimate manuscript.

You may stop me at any time to ask questions, to terminate the recording, or to stop the interview itself. Do you consent to participation in this study? Do you have questions about the interview? May I start the tape recording?

**Demographics**

1. **Please describe your current roles in medical, nursing, or health professions education.** (Interviewer: Check all that apply, confirming that the roles are consistent with the labels below.)

Prompt:

- Are there any educational roles you have held in the past that would be relevant here?
- Dean
- Non-dean administrative position with oversight over educational programs
- Program Director
- Course Director/Leader
- Clerkship Director
- Teaching faculty
- MD, specialty (write in)
- Nursing profession (write in)
- Allied health profession (write in)
- Other (write in)

2. **To clarify, what type of health professional audiences do you predominantly teach?** (Interviewer: Leave it open-ended and check all that apply, confirming if necessary that the roles are consistent with the labels below. If the interviewee has made reference to this topic in question #1, ask “You earlier mentioned that you teach X. Are there any other health professional audiences that you predominantly teach?”)

- Medical students in the preclinical years (1^st^ and 2^nd^ year)
- Medical students in the clinical years (3^rd^ and 4^th^ year)
- Residents
- Fellows
- Practicing physicians/faculty
- Pre-licensure nursing students (BSN or second degree)
- Graduate nursing students
- Practicing nurses
- Advanced practice nurses (e.g., NP, CRNA)
- Other allied health professionals (please specify)
- Other (write in)

3. **What is the total number of years have you been teaching in medical (or nursing or health professions) education?** (Interviewer: write in number of years. Exclude years of teaching during training/residency.)

**4. What is your academic rank?**

- Instructor
- Assistant Professor
- Associate Professor
- Professor
- Other (write in)

5. **I would like to hear about settings in which you teach. After each item, please tell me if you teach in that particular setting.** (Interviewer: Similarly, if the interviewee has already answered this question in an earlier question, confirm and ask if there are any other settings. Check all that apply.)

- Small group setting (e.g., classroom or conference room)
- Large group setting (e.g., lecture hall or conferences)
- Outpatient setting (e.g., clinic)
- Inpatient setting (e.g., hospital)
- Learning center/simulation center
- Other (write in)

**Teaching critical thinking**

6**. You should have received this definition in the email confirming our interview date and time. We defined critical thinking as “the ABILITY to apply higher cognitive skills (e.g., analysis, synthesis, self-reflection, perspective-taking) and/or the DISPOSITION to be deliberate about thinking (being open-minded or intellectually honest) THAT LEADS TO action that is logical and appropriate.”**

Interviewer: If faculty ask how this is different from clinical reasoning, you can say CT is the umbrella term, and clinical reasoning is a form of CT.

**Does this fit with your sense of critical thinking? If not, how would define critical thinking?** (write in)

**Given this definition, can you give me specific examples of how you teach critical thinking?** (write in)

Prompts to flesh out the teaching context

- Make sure you clarify the settting in which the teaching is occurring.
- How do you adjust your strategies in other teaching contexts than the one you just described?
- How do you adjust your strategies with other types of learners?
- This sounds like your primary mode of teaching critical thinking. Do you have other strategies that you use?

Optional prompts if the interviewee seems to have trouble answering:

- Push them to be more specific.
- If not getting a thorough answer, ask them to describe a specific strategy.
- Can you walk me through a time that you were doing this?
- Can you give me a more specific example?
- How do you get the trainee to demonstrate critical thinking?
- If they mention a specific teaching strategy such as PBL, ask them specifically: “Tell me how you think PBL teaches or encourages critical thinking.”

Optional prompts if the interviewee is off topic (not talking about getting LEARNERS to think critically):

- That sounds like you are a good critical thinker. How do you get the LEARNER to think critically?
- This is helpful to hear about the role of your own critical thinking in your teaching process. What would you say your approach is on the learner’s side; that is, how do you get your learners to think critically?

**7. This question asks about more indirect influences on the learners to thinking critically.** **Are there ways in which you motivate or foster critical thinking among individuals without directly teaching it?**

If they seem stumped:

For example, are there activities or strategies that you use when you teach on the fly, prepare for a talk, or run your course? (write in)

8. **How you incorporate critical thinking into the evaluation of students/trainees?**

Optional prompt:

- Can you give me some examples?
- For instance, do you have a portion of your evaluation forms that address critical thinking or some related concept?
- Do you provide specific feedback to trainees about their critical thinking?

**Effectiveness of critical thinking strategies**

9. **Do you have a sense of how well your strategies to teach critical thinking work? Do you ever give formal talks about critical thinking or are you involved in any research about critical thinking?**

Optional prompts:

- Have you ever presented or published about critical thinking? (If so, ask for a citation.)
- Are you conducting a study about critical thinking?
- Anecdotal evidence
- In the midst of study
- Presentation (poster, abstract, or talk)
- Publication (please specify citation)
- Other:

**Conclusion**

That is the end of our questions for you. Do you have any other thoughts related to teaching critical thinking, or is there anything we did not cover that you would like to add?

Thank you so much for participating in this study. We are very grateful for your input. If we have any additional questions about what we covered here, would it be okay if we followed up with you for clarification?

We will be in touch with you about the results of our interviews. We thank you again for your valuable time and contribution.
